# Charge Transfer and Biocompatibility Aspects in Conducting Polymer-Based Enzymatic Biosensors and Biofuel Cells

**DOI:** 10.3390/nano11020371

**Published:** 2021-02-02

**Authors:** Simonas Ramanavicius, Arunas Ramanavicius

**Affiliations:** Department of Physical Chemistry, Faculty of Chemistry and Geosciences, Institute of Chemistry, Vilnius University, Naugarduko 24, LT-03225 Vilnius, Lithuania

**Keywords:** conducting polymers (CPs), electrochemical deposition, microbial and enzymatic biofuel cells, bioelectrochemistry, biosensors, glucose biosensors, polymer-modified electrodes, direct charge transfer, direct electron transfer, electrochemical sensors

## Abstract

Charge transfer (CT) is a very important issue in the design of biosensors and biofuel cells. Some nanomaterials can be applied to facilitate the CT in these bioelectronics-based devices. In this review, we overview some CT mechanisms and/or pathways that are the most frequently established between redox enzymes and electrodes. Facilitation of indirect CT by the application of some nanomaterials is frequently applied in electrochemical enzymatic biosensors and biofuel cells. More sophisticated and still rather rarely observed is direct charge transfer (DCT), which is often addressed as direct electron transfer (DET), therefore, DCT/DET is also targeted and discussed in this review. The application of conducting polymers (CPs) for the immobilization of enzymes and facilitation of charge transfer during the design of biosensors and biofuel cells are overviewed. Significant attention is paid to various ways of synthesis and application of conducting polymers such as polyaniline, polypyrrole, polythiophene poly(3,4-ethylenedioxythiophene). Some DCT/DET mechanisms in CP-based sensors and biosensors are discussed, taking into account that not only charge transfer *via* electrons, but also charge transfer *via* holes can play a crucial role in the design of bioelectronics-based devices. Biocompatibility aspects of CPs, which provides important advantages essential for implantable bioelectronics, are discussed.

## 1. Introduction

Advanced technologies and materials are required to fulfil new challenges that have been raised during the development of analytical systems that are required for food, beverage, environmental, and biomedical analysis. One of the most promising research directions, which is aiming to solve these challenges is related to the development of biosensors. Therefore, nowadays, biosensor-based techniques are applied for the determination of different biologically active materials [1,2]. Amperometric enzyme-based biosensors are the most frequently used among many other types of biosensors [3,4]. Enzymatic and non-enzymatic (enzyme-mimicking) [5] reactions are the most frequently exploited during the action of catalytic biosensors and sensors. Very similar principles can be used in enzymatic [6] and microbial biofuel cells [7,8], which can eventually be applied for a long-lasting electrical current supply for implantable biosensors and some other bioelectronics-based devices [9]. However, during the action of these bioelectronics-based devices, charge transfer from the active site of the redox enzyme is the most critical issue, which limits the generated voltage and current. Direct charge transfer ability can be well exploited for the transfer of electric charge between redox centers of enzymes and electrodes in many bioelectronics-based devices including amperometric biosensors and biofuel cells [10,11,12]. In order to extend the efficiency of charge transfer, inorganic and organic redox mediators [13,14] or additionally added enzyme cofactors [15,16] and/or some types of semiconducting materials [17] can be applied. Some types of redox polymers and redox polymer-based composites can also be applied for this purpose and/or in order to improve the microenvironment required for efficient action of immobilized enzymes or other redox-able proteins [18,19,20,21]. However, from this point of view, conducting polymers seem to be the most promising because they can be used in order to advance charge transfer efficiency in bioelectronics-based devices [22] and some analytical characteristics of biosensors [23,24]. In addition, conducting polymers have great environmental stability [25] and are characterized by rather good biocompatibility [26]. Conducting polymers (CPs) are organic materials, which have rather good electrical conductivity [27,28]. Polypyrrole (Ppy), polyaniline (PANI), and polythiophene (PTH), poly(3,4-ethyle nedioxythiophene) (PEDOT) are mostly used in the design of various high-tech devices and technological applications such as corrosion preventing layers [29], accumulators [30], solar cells [31], super-capacitors [32,33], coatings for electromagnetic shielding [34], sensors [35,36,37,38], and biosensors [39].

Due to the high technological potential of CPs, a lot of attention has been dedicated to the synthesis of these materials, and many chemical [40], electrochemical [39,41], enzyme assisted [24], and even living cell-based CP synthesis approaches have been developed during the last decades. During the here mentioned synthesis of CPs, various structures based on CPs can be designed and the formed CPs can be easily doped by various compounds and ions. In addition, some biological molecules can be entrapped within the formed conducting polymer-based layers and these molecules (e.g., antibodies [42], receptors [39], DNA [43], and enzymes [44]) in many cases can retain some biological functions, which are important for biosensors and/or biofuel cells. If such CP-based composite materials are integrated within proper electronics, they exhibit characteristics that are required for particular bioelectronics. In some studies, it was demonstrated that some CPs are not only compatible with biomolecules and are providing well suitable confinement for these molecules, but are also compatible with neuronal cell lines [45], osteoblastics cells [46], and stem cells [47] and do not irritate the immune system of mammalians [26]. Such good biocompatibility of some conducting polymers provides new possibilities for the application of them as ‘stealth coatings’ during the design of implantable biosensors and biofuel cells. Biofuel cells are based on bio-functionalized electrodes that can generate electrical power using some chemicals that are present in physiological fluids. Biofuel cells can be open for the uptake of these chemicals, which are renewable by metabolic processes, therefore, biofuel cells can use a practically unlimited amount of these materials. Glucose is among the most reliable fuels for implantable biofuel cells [48]. These facts enable significantly reduced dimensions of biofuel cells [49,50]. Hence, the possibility of applying biofuel cells for the powering of biomedical devices seems very attractive because it provides a good balance between the size of a power source and implanted biomedical device (e.g., sensor). Some ‘implantable’ biofuel cells are able to generate electrical power by conversion of glucose and some other chemical compounds, which are present in blood and other fluids of mammalians [51,52,53,54,55], however, the biocompatibility aspects of such biofuel cells still have been not well covered. Therefore, in this review, some insights toward biocompatibility aspects of conducting polymers, which can be applied to hide the most immunogenic parts of implantable biomedical devices from the immune system of the patient, are outlined. Some CPs are finding very interesting applications in the design of sensors based on molecularly imprinted conducting polymers, which can be imprinted by various molecules ranging from rather small organics up to rather large DNA-based structures and even proteins. Such CP-based structures possess rather high sensitivity and selectivity comparable to that of affinity sensors based on immobilized antibodies, receptors, and/or other affinity toward analyte exhibiting molecules/compounds [3,56]. Hence, CPs have many valuable properties that can be well exploited in sensors, biosensors, and biofuel cells.

Therefore, in this review, we aim to overview some the most attractive methods of CP-synthesis, and the involvement of some CPs in the charge transfer between biological structures (enzymes and/or living cells) and electrodes, which is critical in the development of amperometric biosensors and the design of biofuel cells.

## 2. Immobilization of Biomaterials within Conducting Polymer-Based Structures

Different CP synthesis methods were applied for the modification of electrodes applied in the design of amperometric biosensors, which are mainly based on chemical synthesis, electrochemical techniques, and the biochemically induced formation of CP-based structures.

**Figure 1:**
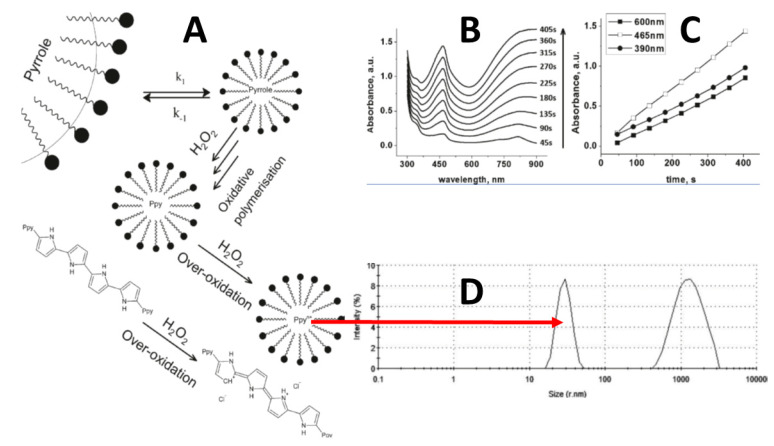
(**A**) Polypyrrole particle formation initiated by H_2_O_2_. (**B**) During the course of the polymerization reaction increasing optical absorbance spectra. (**C**) Increase of optical absorbance at 390, 465, and 600 nm during the formation of Ppy. (**D**) Spectra of formed Ppy diameter determined by dynamic light scattering.

The application of oxidizing compounds in the chemical synthesis of conducting polymers is rather frequently applied to design conducting polymer-based sensing structures. Chemical synthesis of CPs is based on the involvement of rather strong oxidizers such as FeCl_3_ or H_2_O_2_ [40]. A very attractive way that is suitable for the synthesis of some CPs is based on the initiation of their polymerization by H_2_O_2_ (Figure 1A). This method is important because a rather clean conducting polymer can be formed by using H_2_O_2_ because the extent of this compound is easily degrading into water and oxygen and rather clean conducting polymers can be formed. The only drawback of H_2_O_2_-based synthesis is that for the more efficient formation of some CPs, the addition of some surfactants is necessary, therefore, in addition to CP-based structures, some surfactant surrounding the formed particles remains in the solution [40]. In one of our studies, we showed that the formed polypyrrole optical absorbance maximum was ~465 nm and constantly increased during the course of H_2_O_2_ induced polymerization (Figure 1B,C), and finally, polypyrrole particles ~30 nm in diameter formed (Figure 1D) [40].

It was determined that Ppy-based particles have a sufficient biocompatibility with enzymes and even living cells [47,57] and minimally irritate the immune cells of mice while such Ppy-particles were injected into the peritoneum of mice [26]. We have also demonstrated that some CPs (e.g., Ppy) can be synthesized using [Fe(CN)_6_]^4−^/[Fe(CN)_6_]^3−^-based redox cycling [58]. This synthesis route was later applied for the modification of some microorganisms by this conducting polymer [59]. Using chemical synthesis, a large quantity of CP can be formed; mostly, formed CPs are in the form of nano-and micro-particles that remain suspended in colloidal solution and can later be deposited on the surface of the selected electrode. If it is necessary, such particles can be further modified by some other molecules and/or structures. The main drawback of chemically synthesized conducting polymers is not sufficient solubility in traditional solvents, which significantly decreases the processability of formed CPs.

### 2.1. Enzyme Induced Formation of Conducting Polymers

**Figure 2:**
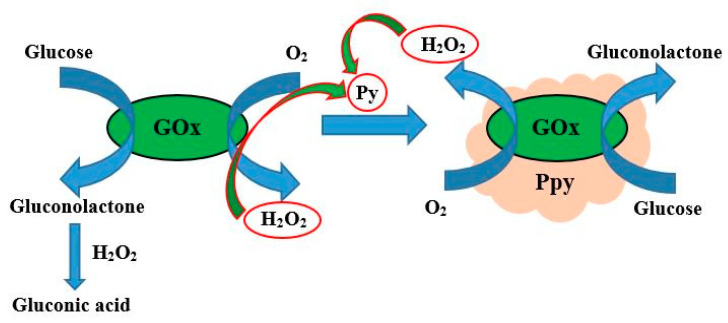
Formation of polypyrrole by glucose oxidase assisted polymerization, according to our researches [24,44,61,64,68,69].

Enzymes, which belong to the class of oxidoreductases, can be successfully applied for the synthesis of some conducting polymers. There are two main routes of enzymatic formation of CP: one is based on the direct formation of a polymerizable species in the active site of enzyme and another one is based on the initiation of the polymerization reaction by redox compounds (e.g., H_2_O_2_), which are formed during enzymatic reaction catalyzed by oxidoreductases [60,61,62,63,64,65,66,67,68,69]. In such a way, the formation of polypyrrole was performed by glucose oxidase (GOx) assisted polymerization and GOx was encapsulated within the formed Ppy layer [24,44,61,64,68,69] (Figure 2).

Such CP formation reactions are performed in the presence of environmentally friendly compounds; therefore, they are often ascribable to ‘green’ chemistry based technologies. Another advantage of such synthesis is that enzymatic formation of CPs can be performed at room temperature and at almost neutral pHs [62,63]. Glucose oxidase (GOx) and some other oxidases act as oxidizers of various substrates, in addition, at natural conditions in the presence of the dissolved oxygen, they generate H_2_O_2_ [24], which is a rather strong oxidant and can induce polymerization of some monomers, namely pyrrole [24,44,61,64,68,69], aniline [61,65], phenanthroline [11], thiophene [61,66], and 9,10-phenanthrenequinone [67], which all by this polymerization method are forming corresponding conducting polymers. Polymerizable monomers can be polymerized by oxidases purified from different microorganisms.

**Figure 3:**
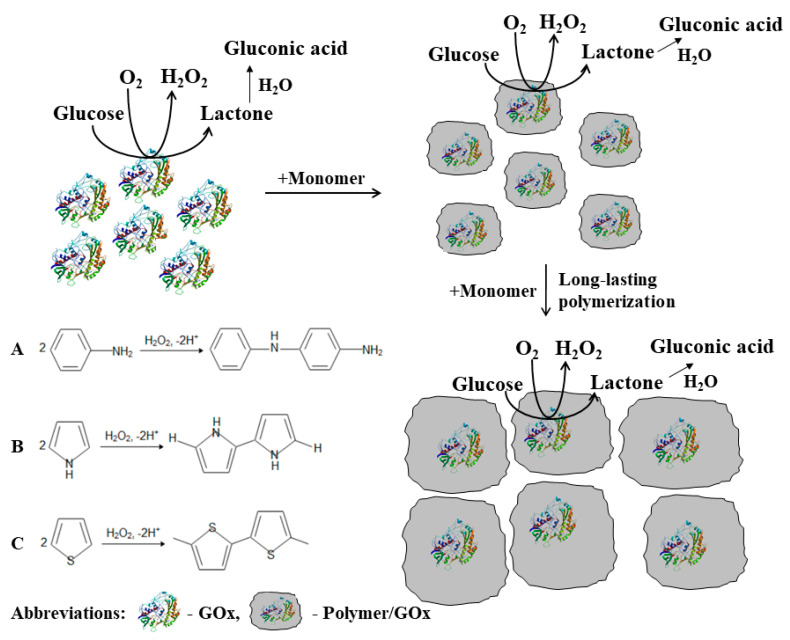
Formation of conducting polymer (**A**—polyaniline, **B**—polypyrrole, **C**—polythiophene) layers around the redox enzyme—glucose oxidase, which during catalytic action is producing H_2_O_2_, which in the presented polymerization reactions acts as an initiator. Adapted from [61].

For the formation of CPs by water dissolved and by immobilized H_2_O_2_, generating enzymes can be applied (Figure 3) and used to tune some analytical characteristics of enzymatic-amperometric biosensors such as apparent Michaelis constant (*K*_M(app.)_), which is extended due to the formation of an additional diffusion layer and an increase in charge transfer efficiency [68,69], which is especially effective if additional structures that facilitate charge transfer through the CP-based layer are embedded within the CP-based layer. The stability of most enzymes is limited [70,71], therefore, in some particular cases, the stability of immobilized enzymes can be improved by ‘self-encapsulation’ of enzymes during enzymatic polymerization of CPs due to rather good biocompatibility of formed CP-layers with entrapped enzymes (e.g., glucose oxidase immobilized on AuNPs/graphite electrodes become at least three times more stable when covered by the Ppy layer) [72]. Therefore, this method is very useful in the design and improvement of amperometric biosensors and design of biofuel cell anodes and/or cathodes, while enzymatic reactions can be applied for the generation of electrical current.

### 2.2. Microorganism-Assisted Synthesis of Conducting Polymers

**Figure 4:**
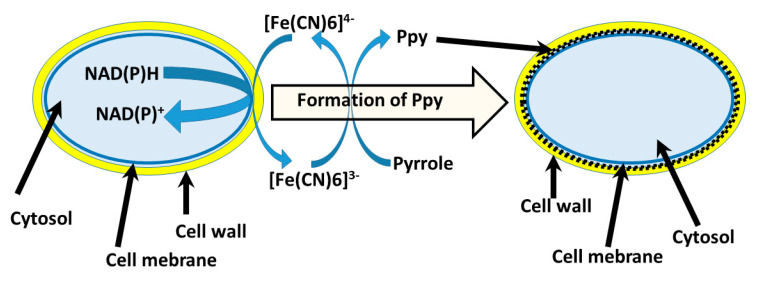
The scheme of Ppy formation in yeast cell wall [59]; Enzymes—oxido-reductases, which are present in plasma membrane, oxidize [Fe(CN)6]^4−^ into [Fe(CN)6]^3−^, which induces the polymerization of pyrrole [58].

Various microorganisms [73,74] and even some mammalian cells (such as erythrocytes [75] and lymphocytes [76]) can be applied in the design of biosensors and biofuel cells, however, charge transfer from these microorganisms toward the electrode is always the key issue and major challenge during the design of these bioelectronics-based devices. The employment of microorganisms in the formation of CPs enables us to improve some charge transfer properties of microorganisms modified in this way. In numerous studies, it was demonstrated that living cells [47,57] and microorganisms [59,77] could retain their biocatalytic properties after modification by conducting polymers. Microorganism-based synthesis of conducting polymers is very advantageous because microorganisms can retain their biocatalytic activity for a much longer period of time in comparison to isolated enzymes [78]. Therefore, various microorganisms have been applied for the formation of different polymers [79] including conducting polymers, e.g.: we have used several types of living microorganisms in the formation of polypyrrole, which is one of the most popular among the recently used conducting polymers. In another related research, we synthesized polypyrrole by ‘redox cycling’ of [Fe(CN)_6_]^4−^/[Fe(CN)_6_]^3−^, which was assisted by metabolic processes running in yeast [59]. As has been reported in our previous research, the formation of Ppy can be induced by [Fe(CN)6]^3−^ [58,59]. Therefore, in the case, if ‘redox cycling’ of [Fe(CN)_6_]^4−^/[Fe(CN)_6_]^3−^ is performed by redox-enzymes—oxido-reductases, which are present in plasma membrane, then Ppy is formed within the cell wall of yeast cells (Figure 4) [59]. Later, we showed that polypyrrole formation can be performed without any additional redox-able compounds [77].

Bacterial strain *Streptomyces* spp., which synthesizes some extracellular redox enzymes including phenol-oxidases, can initiate the polymerization of various phenolic compounds, and was applied for the synthesis of polypyrrole [80]. In order to determine the location of polypyrrole formed within the microorganism, we applied the ‘nonradioactive isotope method’, which showed that polypyrrole was formed within the cell-wall and periplasmic area, which is between the cell-wall and cell-membrane [81]. It is very attractive that after the formation of Ppy, the modified cells remained viable, and synthesized Ppy integrated into the cell wall and in the interphase area between the cell-wall and cell-membrane. In this case, the synthesis of polypyrrole is induced by oxidized products formed during the catalytic cycle of enzymes that are involved in metabolic processes of microorganisms and/or other living cells. We determined that Ppy-based structures form intergrowths within the cell wall of microorganisms (e.g., yeast cells) and, in this way, they influence the elasticity of the cell wall and charge transfer efficiency through the cell wall and membrane [81]. Through this conducting polymer, polypyrrole, the formation method sufficient for charge transfer efficiency through the cell wall was achieved to form Ppy-modified *Rhizoctania* sp. and *Aspergillus niger* [77,82,83], which can be applied in the design of biofuel cells [77]. A similar CP formation method was applied for the enhancement of the cell wall conductivity of *Streptococcus thermophilus*, *Ochrobacterium anthropic*, *Shewanella oneidensis*, and *Escherichia coli* [84]. Increased charge transfer efficiency enabled these microorganisms to be applied in biosensors [85,86] and in microorganism-based biofuel cells (MBFCs) [77]. [Fe(CN)_6_]^4−^/[Fe(CN)_6_]^3−^ conversion based redox cycling enables the formation of Ppy in the solution [58] and inside living cells [59]. In this way, some mammalian cells are also modified by polypyrrole [87]. There are some expectations that some cell lines can be modified by conducting polymers and will probably find practical applicability in biofuel cells.

### 2.3. Electrochemical Synthesis of Conducting Polymers

**Figure 5:**
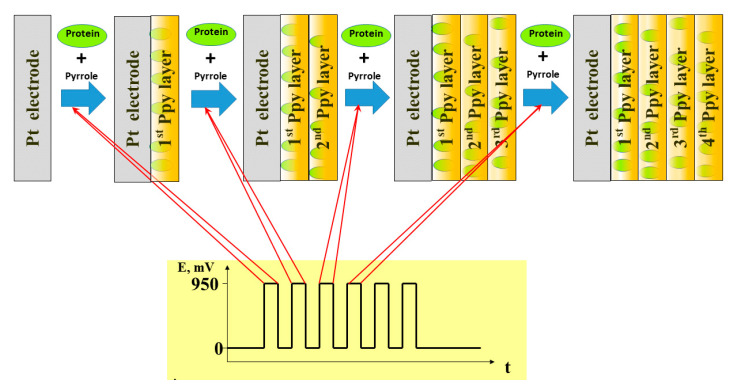
The scheme of Ppy electrochemical deposition by potential pulses and entrapment of proteins within the formed Ppy layer.

Electrochemical synthesis is very efficient during the formation of CP-based layers on the electrode surface. A variety of electrical characteristics should be adjusted in order to form a CP-based layer with the expected physico-chemical characteristics, but the most important among them are: (i) adjustment of the most optimal potentials required for initiation of polymerization reaction, and for periods that are applied between polymerization periods, this is important when potential pulses are applied for the polymerization [39]; (ii) setting up of the limiting current, which is important when galvanostatic approaches are applied, and (iii) potential scan diapason and sweep rate, which is important when potential cycling is applied [25,88]. Hence, the physico-chemical properties (thickness, permeability and some others) of CPs can be controlled by changing these electrical characteristics and some chemical parameters such as composition and pH of the polymerization bulk solution. In addition, various biologically active materials (e.g., proteins) (Figure 5) can be entrapped within the conducting polymer backbone by adding them into the polymerization bulk solution [89,90,91,92].

**Figure 6:**
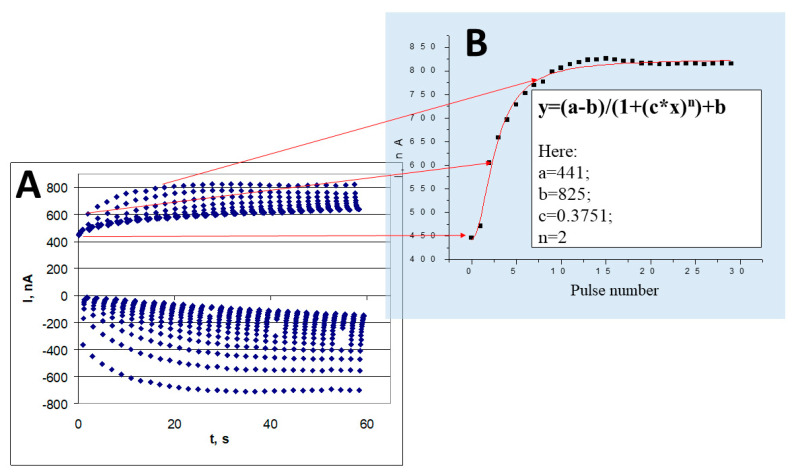
(**A**) Chrono-amperogram, registered during electrochemical deposition of polypyrrole by potential-pulse mode. (**B**) Dependence of anodic peaks on the pulse number during electrochemical deposition. Figure drawn according data presented in [30].

Adjustment of proper electro-polymerization conditions enables the analytical characteristics of CP-based sensors to be changed [24,93]. Electro-deposition of the CP-based layer enables layers to be formed with different characteristics and to cover electrodes by layers with different selectivity and sensitivity. These electrodes can form sensor-arrays that can be applied for the determination of multiple analytes [94]. The diffusion of organic compounds, which acts as an organic fuel of the biofuel cell, *via* a matrix-based on CPs, is also a significant factor during the generation of electrical current by amperometric biosensors and electrodes used in biofuel cell design. Electrochemically accessible surface area and porosity can be changed by the incorporation of organic molecules as spacers between CP-forming chains [95]. The effect of various parameters on the conductivity of free standing electrosynthesized polypyrrole films [96] and formation of polyaniline-based urea biosensors [97] was well analyzed by Lakard’s research team. In addition, we have provided a mathematical model (Figure 6B) suitable for the calculation of the electrochemical formation of polypyrrole by potential pulses [40] (Figure 6A).

## 3. Physicochemical Characteristics of Conducting Polymers

The polymeric backbone of conducting polymers is based on conjugated π–π bonds, therefore, through these bonds, electrical charge can be easily transferred *via* the polymeric chain [92,98,99]. Due to advanced conductivity and other attractive charge transfer properties [100,101], CPs are applied in the design of light emitting diodes, monitors, batteries, sensors, and organic-based photovoltaic devices and smart windows, which can be installed in cars, houses, and some other infrastructural units [102,103,104]. In addition to unique electrical properties [24], some structures based on CPs can show very selective affinity [105] and/or advanced optical/spectral characteristics [106]. Hence, the variation of some of the physical characteristics (e.g., changes of electrical impedance and/or capacitance, variation of spectral characteristics or fluorescence behavior, etc.) of the sensing layer based on conducting polymers is mostly exploited for the registration of analytical signal. A vast number of conducting polymers has been used in the structure of amperometric biosensors and biofuel cells, but among them, polypyrrole is used the most frequently [24]. Some conducting polymers form porous [107,108] and/or gel-based structures [109,110,111], therefore, they are well suited for the efficient immobilization of redox enzymes that need water for their catalytic activity, which is required for amperometric biosensors and biofuel cells. Furthermore, some conducting polymers possess a rather low solubility in water, but they have been reported as biodegradable, therefore, such CPs can be exploited in the design of biodegradable electronics and/or bioelectronics [112,113]. Some CP-based composite materials are selective to particular metal ions [114], hence, they can be applied in the development of analytical systems for the detection of mercury(II) [115], lead(II) [115], and copper(II) [115,116].

## 4. Compatibility of Conducting Polymers with Proteins, Living Cells and Immune System of Mammalians

Nowadays, implantable biomedical devices are very rapidly evolving [117] and they demand miniature power sources [118]. Therefore, the demand for biofuel cells suitable for implantable bioelectronics-based devices, especially for biosensors, is constantly increasing. However, this research direction has many specific challenges [119], one of which is related to various biocompatibility aspects of implanted biosensor and/or biofuel cell structures, which can be fouled by proteins and/or other biomolecules [120,121,122], which are present in various ‘body-fluids’, and/or can irritate the immune system of the patient [123]. Despite these challenges, biofuel cells are ‘occupying new horizons’ including implantation into various plants [124,125] and organisms (including rats [126,127,128], rabbits [129], snails [53], clams [55], and insects [130]), and despite the divergence of many different opinions [131,132], they are expected to be successfully implanted in the human body [133,134,135].

Many various researches have been dedicated to evaluate some biocompatibility aspects of CPs with proteins [3,11,24,61,64], DNA [43,105], and stem cells [47,57] and microorganisms [59,81,82,83]. However, only a few of them have been dedicated to investigate how conducting polymers affect the immune system of mammalians [26]. These biocompatibility-related issues have become the most important because some biofuel cells and amperometric biosensors have recently been implanted into patient organs [136,137] or attached to different parts of the body (e.g., to skin, eyes, mucosa) [138]. If the biocompatibility of implanted/attached biosensors and/or biofuel cells [139] with the patient body is not sufficient, then inflammation and various forms of allergies can be induced [140,141]. Selection of a proper immobilization method suitable to retain the activity of immobilized biomaterial is critical during the development of biosensors [142,143] and biofuel cells. Therefore, many studies have been dedicated to the assessment of CP-compatibility with proteins, and here, practically all cases where entrapped, covalently immobilized, and/or adsorbed proteins retained their biological functions can be declared as biocompatible. In this research direction, we evaluated the influence of polypyrrole toward more advanced ‘biological systems’ such as living stem cells [47,57] and or the immune system of mammalians [26]. In the last here mentioned research, we determined that polypyrrole does not has any significant effect on the immune system of mice cells because these hematological parameters, which reflect the state of the immune system, remained unchanged [26]. However, some dose-dependent influence of polypyrrole-based nanoparticles on bone marrow-derived stem cells has been observed at a rather high concentration of nanoparticles [57]; here if a low concentration of polypyrrole nanoparticles was applied, the toxic effect to mouse hepatoma (MH-22A), human T lymphocyte Jurkat, and primary mouse embryonic fibroblast (MEF) cells was not observable [57]. Above-mentioned evaluations illustrated that polypyrrole is rather well biocompatible with assessed cell-lines [47,57] and are compatible with the immune system of mammalians (laboratory mice) [26]. Some biocompatibility related aspects of the conducting polymer polyaniline were also evaluated and determined [144]. Moreover, in some scientific works, it was demonstrated that some specific stimulation by an electric field induced nerve cell differentiation deposited on a composite structure consisting of polypyrrole/poly(2-methoxy-5 aniline sulfonic acid) [145]. There are some positive expectations that the biocompatibility of CP-modified electrodes can be increased when they can be mixed with some other biocompatible polymers (such as chitosan [146,147,148]) and/or form hydrogels that contain a significant amount of water [109,149,150]. Such conducting polymer-based gels can be applied as the scaffolds for the incorporation of some tissue-forming cells [151,152], which can be used for tissue engineering and/or transplantation [153] as well as in many other fields of biomedicine [154,155,156,157]. The rather good biocompatibility of polypyrrole and some other conducting polymers enables the use of these polymers in the creation of enzymatic biofuel cells [77] that can power some implantable/attachable sensors or other biomedical tools. However, it should be noted that the number of real biocompatibility-based evaluations is still not very high, therefore, significant attention could be paid to this research direction.

## 5. Most Important Functions of Conducting Polymers in Amperometric Biosensors and Biofuel Cells

**Figure 7:**
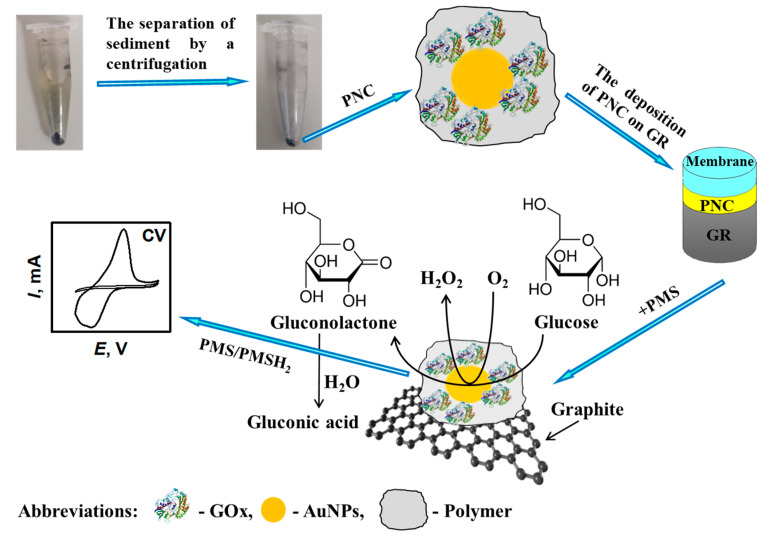
Principle scheme of the formation of a composite structure consisting of polyaniline (PANI), gold nanoparticles (AuNPs), and glucose oxidase (GOx) PANI/AuNPs-GOx, which is followed by a cyclic voltammetry-based investigation. Adapted from [168].

Amperometric biosensors and biofuel cells are mostly based on immobilized enzymes or living cells [158,159]. Among the many oxidoreductases, glucose oxidase (GOx) is used mostly in biosensor design [160]. The same GOx can be well applied for the development of biofuel cells [161,162,163] and self-charging capacitors [164] based on the operation of biofuel cells [165,166,167]. GOx itself can be involved in the polymerization reaction of many CPs, namely, polypyrrole, polyaniline (Figure 7), polythiophene, etc.

CPs-based layers play a number of different roles in the design of amperometric sensors because they can serve: (i) as an immobilization matrix [24]; (ii) as a diffusional barrier for enzymatic reaction substrate, which increases the so called apparent Michaelis constant (*K*_M(app.)_) for immobilized enzymes and, therefore, in this way can extend the linear-range of amperometric biosensors [44]; and (iii) and in some cases, they act as charge transfer mediators [11]. Therefore, the entrapment of enzymes within conducting polymer-based structures enables some bioanalytical characteristics (such as limits of detection and linear ranges) of biosensing systems to be changed. Biosensors based on GOx, which is modified by conducting polymers (e.g., polyaniline, polypyrrole, or polythiophene) have been reported and in such systems, soluble redox mediators (ferrocene, benzoquinone, 2,6-dichlorophenol indophenol, phenazine methosulfate, and some others) were applied in order to facilitate charge transfer between the enzyme and electrode. Facilitation of indirect CT by the application of some nanomaterials (such as metal and semiconductor nanoparticles) is rather simple, therefore it is applied in most electrochemical enzymatic biosensors [65,68,69] and biofuel cells [169].

**Figure 8:**
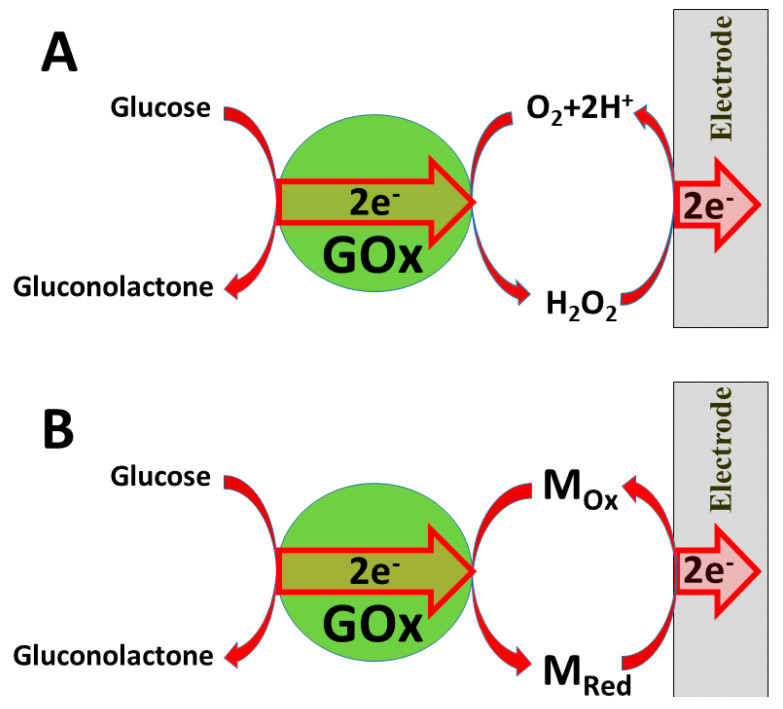
Charge transfer from glucose oxidase (GOx): (**A**) *via* formed hydrogen peroxide; (**B**) *via* redox mediator M_ox_/M_Red_.

Several ‘generations’ of amperometric biosensors are determined according to the applied charge principle. In ‘first-generation’ amperometric biosensors, charge is transferred via enzymatic reaction products [170] (e.g., if oxidases are applied, then electrons from one substrate are transferred to dissolved oxygen and hydrogen peroxide is formed [171]); in the case of such amperometric sensors, the analytical signal can be based on electrochemical registration of decreasing oxygen concentration or increasing hydrogen peroxide concentration (Figure 8A).

**Figure 9:**
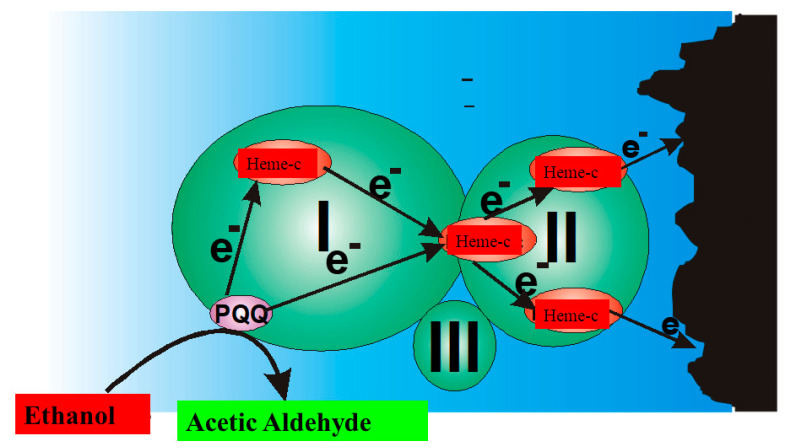
Charge transfer within PQQ-Heme dependent alcohol dehydrogenase, and direct electron transfer from PQQ-Heme dependent alcohol dehydrogenase, which can be applied in the design of biofuel cells [161] and amperommetric biosensors of the third generation.

In the ‘second generation’ of amperometric biosensors, dissolved charge transfer mediators are applied that transfer the charge while oxidized/reduced forms of these redox mediators diffuse between the redox-able active site of the enzyme to the electrode (Figure 8B). In the ‘third generation’ of amperometric biosensors, charge transfer is based on the direct exchange of charge carriers between the enzyme’s active site and electrode [172,173], and the same effect can be exploited in direct electron transfer-based biofuel cells [161] (Figure 9); to improve/facilitate this process, conducting polymers can be applied [174,175,176]. Sometimes, additional sophisticated ‘wiring’ routes are applied in order to establish the charge transfer between the redox sites of enzymes and electrodes [174,177,178,179].

It should be noted that fast and efficient charge transfer is especially critical for the action of biofuel cells. It should be noted that in addition to the charge transfer between the enzyme and electrode, very critical is the understanding of the charge carrier pathways and their dynamics within oxidoreductases applied in the design of bioelectronics-based devices [179]. From the scientific point of view, understanding of the charge transfer pathways and mechanisms is extremely important in order to exploit enzymes efficiently [180], which is important during the design of biofuel cells and biosensors. In some redox enzymes, charge transfer pathways are rather complex because some radicals of amino acids can be involved in intrinsic charge transfer pathways [181]. Such amino acids (e.g., tryptophan and tyrosine [182]) are mostly based on aromatic radicals and they can attend not only in intrinsic, but also in extrinsic charge transfer pathways that are typical for various biological systems [183,184,185]. Both electron- and hole-based charge transfer pathways can be observed between some redox enzymes (e.g., in GOx) and some conducting polymers that act as p-type organic semiconductors (e.g., carbazole-derivatives) [174,179].

Some other p-type semiconducting polymers (including poly(3,4-ethylenedioxythiophene (PEDOT) due to suitable ionization potential, which is below 5.0 eV) show sufficient ability to transfer holes [186]. It was predicted that charge transfer *via* hole hopping to some extent protects enzymes from oxidative damages [187]. Such polymers are able to not only transfer charge via holes, but can even inject them into the intrinsic charge transfer pathway of some redox enzymes including glucose oxidase as reported for some carbazole derivatives [174,179] or PEDOT [188,189,190]. Application of such p-type semiconducting polymers is very promising for biosensors and biofuel cells because it enables the stability of the enzymes to be retained for a longer period of time, therefore, in some of our works, we applied several p-type semiconducting carbazole-based derivates for the development of rather stable glucose oxidase-based biosensors [174,179]. The action of such glucose biosensors is well supported by DFT-based computations [179], which enabled the charge transfer mechanism to be elaborated not only in polymer, but also inside the enzyme, and to calculate charge transfer characteristics [179] that were in agreement with those determined by experimental approaches [174]. All these properties of conducting polymers can be applied during the development of advanced biosensors, which will have analytical characteristics better suitable for particular analytical purposes (e.g., the entrapment of redox enzymes), which initially possess rather low *K*_M(app.)_, within CPs enables the increase in the ‘upper limits of analyte determination’ due to the formed CP-based ‘diffusion layer’ [24,65,66]. In this way, glucose biosensors can be based on glucose oxidases that mostly have rather low *K*_M(app.)_, which are mostly much lower than the glucose concentration in the blood serum [191].

Some CPs can facilitate electron transfer between the active-site of the enzyme and electrode [11], which is important during the development of biofuel cells and amperometric biosensors [24,44]. However, active-sites in some redox enzymes are located within the protein backbone. Therefore, charge transfer to/from these active-sites is not possible even through conducting polymer-based structures.

**Figure 10:**
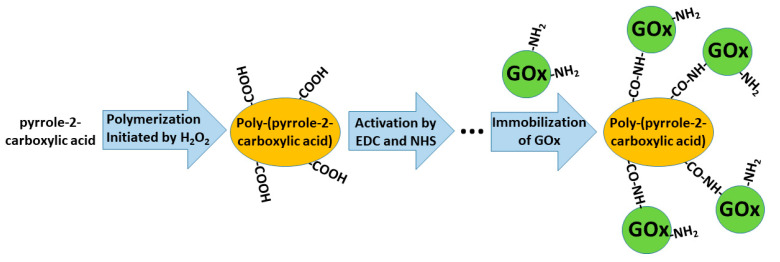
Formation of poly-(pyrrole-2-carboxylic acid), followed by the activation of carboxylic groups and covalent immobilization of glucose oxidase (GOx).

In some of our previously published studies, we determined that charge transfer could be established by structures based on polyphenontraline [11] and carbazole-based derivatives [174,179]. During the modeling of amperometric biosensors, glucose oxidase is applied as a model enzyme. Therefore, glucose oxidase was entrapped within some CPs [11,24,44]. However, electron transfer from the active-site of enzymes and the electrode still remains a challenging problem in these structures, the most frequent charge transfer is established by dissolved redox mediators or by electrodeposited conducting polymers [11]. Therefore, in some biosensors, conducting polymers can serve as electron transfer mediators and as a matrix within which redox enzymes are immobilized [192,193]. The applicability of conducting polymers can be improved by the formation of various copolymers based on monomers that form conducting polymers (e.g., in this way, specific functional groups, which are required for covalent immobilization of enzymes (namely, carboxylic groups, amino groups, etc.), can be introduced) [194]. In this way, the pyrrole-2-carboxylic acid was polymerized into particles of poly-(pyrrole-2-carboxylic acid) (PCPy) by chemical polymerization initiated by H_2_O_2_, and then covalently modified by glucose oxidase *via* formed amide bonds, which were formed after the activation of carboxylic groups by N-(3-dimethylaminopropyl)-N’-ethylcarbodiimide hydrochloride (EDC) and N-hydroxysuccinimide (NHS). During this activation step, EDC reacts with carboxyl groups and forms active O-acylisourea intermediates, which couple NHS and form amine-reactive N-sulfosuccinimidyl esters on the surface of the PCPy layer that during the next step react with the amino groups of glucose oxidase. Then, this GOx/PCPy nanocomposite was applied for the modification of graphite electrodes and applied in the design of a glucose sensor [194] (Figure 10).

The immobilization of enzymes enables biosensors to be applied for continuous and/or repeating measurements of analytes, however, despite numerous efforts to retain the stable activity of enzymes, they gradually lose their activity, which negatively influences the accuracy of the analytical signal [168]. Hence, limited stability of amperometric biosensors is a drawback that requires special attention and, therefore, these biosensors require additional calibration procedures that are performed periodically and/or periodical exchange/replacement of enzyme-based structures.

It should be noted that various charge transfer reactions play a very important role in photosynthesis, metabolic pathways, and many other biological and artificial redox systems [195,196,197,198]. Both electron and hole transfer mechanisms are important for charge transfer in biosensors and biofuel cells. However, recent developments in electrochemistry and bioelectronics mostly take into consideration only the electron transfer-based reactions. For this reason, advanced understanding of the charge transfer mechanisms and pathways is required for the development of advanced bioelectronics-based devices. In addition, charge hopping and/or tunneling mechanisms [199,200] can be involved for the charge transfer between electrodes and redox enzymes or other redox proteins [201,202]. These mechanisms provide the ability to transfer charge through rather long distances, however, the efficiency of these charge transfer mechanisms is not very high and is always determined by the electrical potential of electrodes and redox potentials of used materials. Conducting polymers, which are used for the modification of electrodes applied in the design of amperometric biosensors and/or biofuel cells, can provide hole- or electron-based conductivity. Therefore, charge transfer between these conducting polymers and redox enzymes could also be evaluated in such a way that takes into account the many different mechanisms of charge transfer between redox enzymes and conducting polymers [174,179].

## 6. Conclusions

Efficient charge transfer (CT) plays a crucial role in the design of biosensors and especially in the development of reliable biofuel cells. In order to improve CT in these bioelectronics-based devices, some nanomaterials are applied. Indirect CT is the most frequently exploited during the design of biosensors and even during the development of biofuel cells, here, nanomaterials, especially metal and semiconductor nanostructures, play an important role. Nanocomposites based on conducting polymers and some nanomaterials can provide various technological advantages including increased surface area, which is required for the establishment of higher current densities that are of special interest during the development of biofuel cells. In addition, CPs offer very attractive ways for the immobilization of enzymes. Among the many different methods used for the formation of conducting polymer-based structures and nanostructures, electrochemical deposition is one of the most efficient due to the possibility of precise control of the polymerization process by the adjustment of the most suitable electrochemical parameters. In addition to electrochemical synthesis, oxidizing agents, redox enzyme, and microbes can be applied as initiators of CP synthesis, which all confirmed that through these ways, formed CP-based enzyme/CP composites could be applied in the design of biosensors and biofuel cells. In enzymatic-electrochemical biosensors and biofuel cells, some other characteristics of the enzyme/CP-based layer are also very important, namely, density, permeability, and thickness of the structure, which is formed over the electrode.

Direct charge transfer (DCT), which is very often simply called direct electron transfer (DET), from enzymes, except DCT/DET for a few types of enzymes (hemoproteins, Cu ion-based proteins, and some other enzymes), is still very rarely realized. Despite of this evaluation of DCT/DET is a very interesting and promissing research direction, which in the future will provide many interesting solutions. Recently, some rather sophisticated DCT/DET pathways from enzymes toward electrodes have been established by several research groups. In some of these DCT/DET routes, conducting polymers (CPs) such as polypyrrole and carbazole-derivatives were applied. In some of our research and theoretical calculations, we have demonstrated that not only electron transfer, but also hole transfer, can play a role and can be well exploited in the design of bioelectronics-based devices.

The good biocompatibility of some conducting polymers provides new possibilities for their application as ‘stealth coatings’ in the design of implantable biosensors and biofuel cells.

## Data Availability

Data sharing not applicable.
